# Hybrid organic–inorganic structures trigger the formation of primitive cell-like compartments

**DOI:** 10.1073/pnas.2300491120

**Published:** 2023-08-10

**Authors:** Silvia Holler, Stuart Bartlett, Richard J. G. Löffler, Federica Casiraghi, Claro Ignacio Sainz Diaz, Julyan H. E. Cartwright, Martin M. Hanczyc

**Affiliations:** ^a^Cellular, Computational and Integrative Biology Department, Laboratory for Artificial Biology, University of Trento, Povo38123, Italy; ^b^Division of Geological and Planetary Sciences, California Institute of Technology, Pasadena, CA91125; ^c^Instituto Andaluz de Ciencias de la Tierra, Consejo Superior de Investigaciones Cientificas–Universidad de Granada, Armilla, Granada18100, Spain; ^d^Instituto Carlos I de Física Teórica y Computacional, Universidad de Granada, Granada18071, Spain; ^e^Chemical and Biological Engineering, University of New Mexico, Albuquerque, NM87106

**Keywords:** origin of life, vesicles, inorganic mineral surfaces, surface chemistry, alkaline hydrothermal vents

## Abstract

Alkaline hydrothermal vents have become a candidate setting for the origins of life on Earth and beyond. This is due to several key features including the presence of gradients of temperature, redox potential, pH, the availability of inorganic minerals, and the existence of a network of inorganic pore spaces that could have served as primitive compartments. Chemical gardens have long been used as experimental proxies for hydrothermal vents. This paper investigates a set of prebiotic interactions between such inorganic structures and fatty alcohols. The integration of a medium-chain fatty alcohol, decanol, within these inorganic minerals, produced a range of emergent 3 dimensions structures at both macroscopic and microscopic scales. Fatty alcohols can be considered plausible prebiotic amphiphiles that might have assisted the formation of protocellular structures such as vesicles. The experiments presented herein show that neither chemical gardens nor decanol alone promote vesicle formation, but chemical gardens grown in the presence of decanol, which is then integrated into inorganic mineral structures, support vesicle formation. These observations suggest that the interaction of fatty alcohols and inorganic mineral structures could have played an important role in the emergence of protocells, yielding support for the evolution of living cells.

## Significance

Hydrothermal vents have long been suggested as an ideal location where abiogenesis could have occurred. Simultaneously, a large volume of research has explored how the first cell membranes may have arisen, leading to the evolution of the first living cells. The present study demonstrates the integration of these two parallel bodies of work in the origins of life. The inclusion of organic molecules in vent analog systems led to the creation of new and unique 3D surfaces, which support the self-assembly of vesicles. This demonstrates that the presence of organic chemistry can modify the inorganic surface architectures, which together lead to the promotion of primitive cell-like compartments.

One of the leading models for the emergence of life on earth is the alkaline hydrothermal vent theory (1–5). Off-axis hydrothermal vents (comparable to the well-known Lost City vent field) are focusing centers for various thermodynamic gradients that can drive a wide range of chemical processes (6–9). Cold, CO2-rich, oxidized sea water as present in the Hadean (10–12), percolates into the porous, mineral environs of the vents. Within the vent chimneys lie networks of microscopic pores, through which the fluid is permeating (3). The seawater eventually circles back to rejoin the ocean, passing through the growing vent chimneys and emerging very different in composition and temperature (13–15). Accordingly to the alkaline vent theory, an array of prebiotic reactions, exploiting disequilibria and the presence of small-molecule precursors took place, producing biomolecules such as small peptides (16–20). In the meantime, the rock-water reaction known as serpentinization could have released H2 in the midst of such vent structures, enabling reducing reactions such as alkane polymerization (3) and ketone reduction to alcohols (21). We can surmise that the synthesis of many prebiotic molecules would have occurred in the presence of hydrothermal vents, that created favorable conditions for the emergence of primitive biochemistry (9, 22, 23).

Alongside molecular synthesis, a key question is the spatial organization (if any) of these complex distributions of compounds. In fact, vent scenarios provide natural mechanisms by which prebiotic molecules could have self-organized toward the first compartmentalized protocells (24, 25), and recent work by Jordan et al. (5, 26) demonstrated the stable self-assembly of protocellular structures under hydrothermal conditions. In contemporary research on protocells, fatty acids are a primary focus (27, 28), and it was demonstrated that RNA can be encapsulated (29, 30) and copied (31) inside fatty acid vesicles. Furthermore, in the context of origins of life scenarios, the stabilizers of early phosphate amphiphile membranes were likely mixtures of mid- to long-chain fatty acids and alcohols (32–34), and medium- to long-chain alcohols have been shown to increase the stability of primitive membranes (35–39). Hydrothermal vents are also likely to have been a source of fatty acids and medium-chain alcohols derived from alkanes (40, 41).

This growing body of results is suggestive of a key role for fatty alcohols and their interactions with fatty acids in hydrothermal vent origin scenarios. In this work, we will focus specifically on one fatty alcohol and three fatty acids in order to keep the system sufficiently simple with a manageable set of control parameters. In particular, we endeavored to explore whether a medium-chain fatty alcohol could be integrated into inorganic mineral vent systems. Chemical gardens have a widely cited role as an effective proxy for laboratory studies in this area; thus, we employed this system in the present work. Commonly used prototypes of Lost City vent field include aragonite, brucite, calcite, and silicate (42–44). Given the presence of calcium carbonate and silicate minerals in Lost City Vents, we chose to reproduce calcium silicate gardens.

As an amphiphilic, 10-carbon chain alcohol, decanol is known to be a weak surfactant with low solubility in water (45). It is the shortest chain length alcohol capable of phase separation in the presence of water and has sufficient chain length to incorporate into fatty acid bilayer membranes and modify their properties without destroying them (35). These properties make decanol an interesting candidate to study as a potential component in prebiotic membranes. Given the plausible presence and importance of these alcohol molecules in ancient alkaline vent scenarios, we analyzed the feasibility of interactions between decanol, alkaline hydrothermal vents, and fatty acids using chemical gardens as a proxy. A range of emergent structures, at both microscopic and macroscopic scales, were observed, and fatty acid vesicle formation was triggered only in the presence of the crystal garden grown in the presence of decanol.

## 1. Results

### A. Macroscopic and Microscopic Effects of Decanol on Chemical Gardens: Vertical and Horizontal Systems.

Macroscopic alterations of two main variants of the chemical garden were grown and observed in vertical and horizontal systems. The systems studied are CG in the case of a typical chemical garden and CGD in the case of the chemical garden grown in the presence of decanol. Decanol was added in CGD systems previously to CaCl2 addition.

**Fig. 1.**
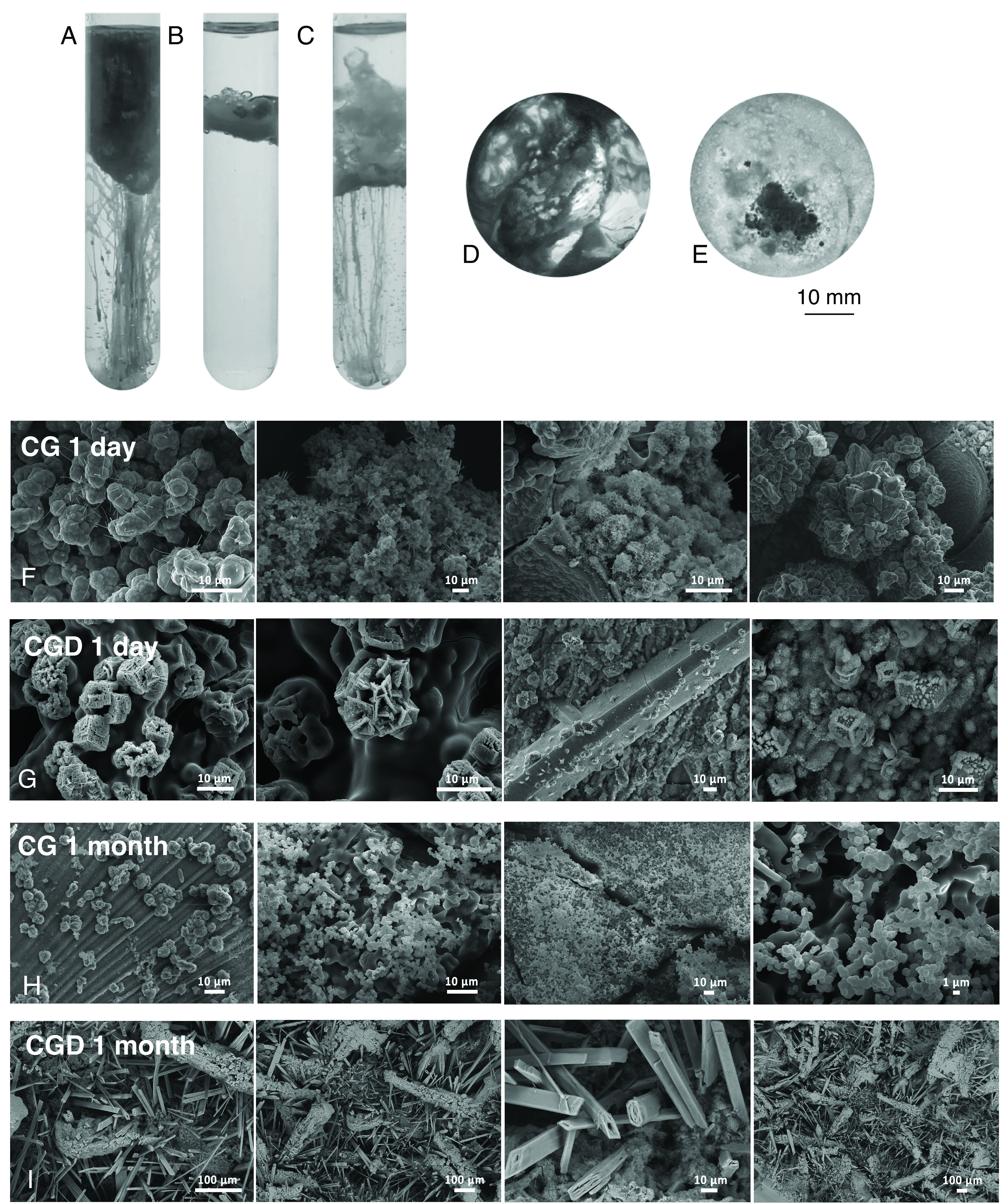
Vertical and horizontal chemical garden (CG) and variants with decanol added (CGD). In (*A*) CG: Calcium chloride seeds were dropped upon sodium silicate solution. In (*B*) CGD, calcium chloride seeds were dropped onto a decanol–silicate two-phase system. In (*C*) CGD after mixing, calcium chloride seeds dropped upon a decanol–silicate mixed two-phase system. In (*D*) horizontal variant of the CG system and (*E*) horizontal variant of the CGD system. Scanning electron microscopy images of samples after 1 d (CG 1 (*F*) and CGD 1 (*G*)) and 1 mo (CG 1 (*H*) and CGD 1 (*I*)).

Calcium chloride seeds when added to a sodium silicate solution fall to the bottom of the glass vial where the silicate solution is previously loaded and create chemical gardens that grow from the seeds upward (Fig. 1*A*, CG). Fig. 1 *B* and *C* (CGD) show two alternative systems where 1-decanol is added to the sodium silicate solution. In Fig. 1*B*, decanol is gently layered on top of the silicate solution, and CaCl2 seeds are dropped upon it. The seeds tend to stay at the decanol–silicate interface where they form a solid barrier. In Fig. 1*C*, decanol and silicate are vigorously shaken before CaCl2 seeds are dropped upon the created emulsion. Some seeds became trapped at the interface, some fell to the bottom of the tube or stopped part way through the decanol silicate emulsion, depending on the seed’s weight and on the density gradient. In both systems with decanol, the seeds form distinct macroscopic structures, varying between one another and in comparison with the CG system. The CGD structures resemble ceiling-like barriers and web-like structures (*SI Appendix*, Video S1).

Further analysis of the structures was performed in a horizontal observation system. Sodium silicate solution was placed in each well of a 6-well plate. Decanol was added to three of the wells, and calcium chloride powder was added manually to all 6 wells. Examples of the obtained systems are represented in Fig. 1*D* (CG) and *E* (CGD). The evolving structures in these two different systems (CG & CGD) look markedly different (*SI Appendix*, Video S2). The crystals localized at the interface between silicate solution and decanol in CGD systems showed random motion. We report in *SI Appendix* a pixel intensity plot that portrays the typical observational differences between CG and CGD horizontal systems (*SI Appendix*, Fig. S1). We show that CGD systems are macroscopically different from CG systems using optical images, video analysis, and pixel analysis. In the CG system (bottom row, Video 2), the calcium chloride powder fell to the bottom of the well and began to generate a chemical garden, starting from where it was dropped and with the auxiliary help of the well’s wall interface. In CGD systems (top row), some calcium chloride powder was trapped at the interface, and some fell to the bottom of the well. The powder trapped at the interface showed some random motion, and the powder that reached the bottom of the well slowly reacted. We then shifted to the microscopic scale and analyzed CG and CGD systems after 1 d and after 1 mo of incubation using scanning electron microscopy (SEM). After 1 d and after 1 mo, the decanol phase was still clearly phase separated from the silicate water phase and transparent. There are clear differences between structures formed without (Fig. 1*F*) and with decanol (Fig. 1*G*) over 1 d. Samples formed without decanol are similar to those typically observed in chemical gardens, mainly composed of a sodium silicate matrix with garden-like features as well as spike-like structures. Samples incubated with decanol instead show more orthogonal structures with some crystals and rosette formations. Systems incubated for 1 mo (Fig. 1 *H* and *I*) show some similar variations and structures to those incubated for 1 d (Fig. 1 *F* and *G*). As above, samples left to react without decanol evolved similarly to typical chemical gardens, and metal ion jets likely created the observable small bubble-like structures (Fig. 1*H*). In contrast, the systems incubated with decanol produced squared protrusions and rectangular structures, demonstrating the long-term influence of decanol addition on the microscopic structure of chemical gardens (Fig. 1*I*). Thus, we deduce that on the microscopic scale, decanol strongly influences the morphology of the mineral inorganic surfaces created from the incubation of sodium silicate with calcium chloride.

### B. Chemical Composition Effects of Decanol on Chemical Gardens: X-ray Photoelectron Spectroscopy and Scanning Electron Microscopy Coupled with Energy Dispersive X-ray Spectroscopy.

After scanning these diverse structures using SEM, X-ray photoelectron spectroscopy (XPS) was used to further analyze the inorganic mineral surface composition. Three samples of type CG and three samples of type CGD were analyzed. Samples were left to react for 1 d before analysis.

**Fig. 2.**
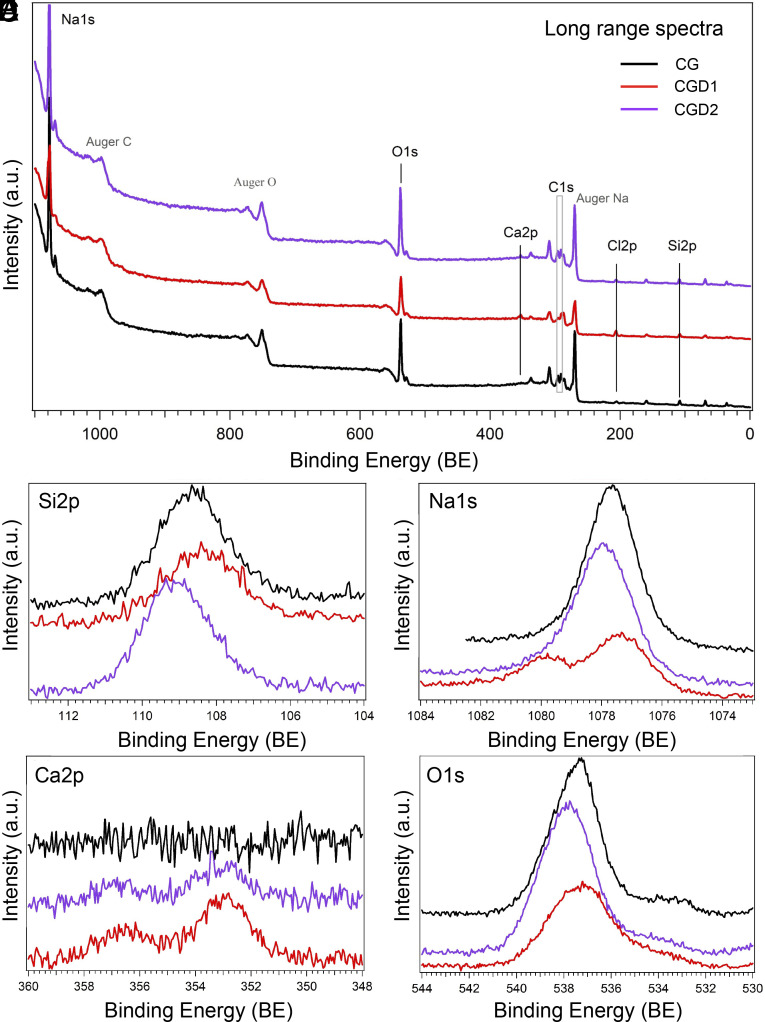
Long-range XPS spectra for samples CGs and CGDs (low resolution, PE = 50 eV) in *A*. Short-range spectra for (*B*) Si2p, (*C*) Na1s, (*D*) Ca2p, and (*E*) 01s. Spectra are not normalized in intensity.

We first analyzed the overall crystal surface composition using long-range XPS spectra. The long-range XPS spectra in Fig. 2*A* show the presence of sodium (Na1s), silicon (Si2p), oxygen (O1s), chlorine (Cl2p), carbon (C1s), and calcium (Ca2p). This analysis shows expected compositions for all the samples. All of them are characterized by one Na1s thin peak from Na-O bonds, and one broad peak in the Si2p profile at 108.45 eV attributed to Si–O bonds (102 eV) and Si–O–Si bonds (103 eV), typical of sodium silicates (46). The O1s profile is characterized by an asymmetric band and two types of oxygen in the silicate matrix: bridging O atoms (BO) connecting two Si oxide tetrahedra covalently and nonbonding O atoms (NBO) bonded to Si but also ionically connected to another cation (in this case, Na or Ca). Furthermore, lineshape fitting was optimized accounting for the contribution of a third component in the deconvolution analysis, with an energy region typical of M-OH bonds (lineshape fitting for O1s can be found in *SI Appendix*, Fig. S2*B*). All these contributions confirm the presence of sodium silicate in our samples.

Moving to high-resolution short-range spectra, we noticed that Na1s core level analysis of CG (two out of three) and CGD (one) samples show the second Na1s high BE peak that can be correlated with the presence of NaCl. In addition, the short-range analysis for the Cl2p core level showed clearly also the presence of CaCl2 in these samples. Furthermore, Na1s, Cl2p, and Ca2p are the three species shown in two distinct peaks for samples CGD1, CG2, and CG3. We can deduce that this is related to the formation of NaCl and CaCl2 aggregates embedded into the silicate matrix of CGs and CGDs. C1s core level analysis is reported in *SI Appendix*, Fig. S2*A*. The energy region where the C1s peak is expected is superimposed on the complex Na Auger emission. However, comparing CGs and CGDs through BE alignment, we highlight some differences between these two surfaces. Two CGDs show a more pronounced lower binding energy shoulder where the C1s core level should be located (“expected C1s range” zone) and an increased level of carbon in these samples can be hypothesized. From this analysis, we conclude that the decanol is incorporated in the CGDs structures albeit in various ways and with different kinetics, varying its quantity in different CGD crystal regions.

In light of these observations, we chose to analyze the CG and CGD systems incubated also over a longer period (1 mo). We used a technique enabling a more in-depth surface analysis: scanning electron microscopy coupled with energy-dispersive X-ray spectroscopy (SEM EDXS). Relevant results are discussed here and corresponding figures can be found in (*SI Appendix*, Fig. S3). Inorganic mineral surfaces created after 1 d of incubation with CaCl2 with or without decanol show an analogous overall composition, similar to the one observed through XPS. Inorganic mineral surfaces generated after 1 mo of incubation without or with decanol also show an overall similar composition with an increased amount of Cl and Ca. In the case of the sample incubated without decanol, a slight silicon presence is noted that is instead absent in the case of decanol treatment. We presume a higher substitution of chlorine ions to silicate and an increased thickness of the salt crystals present on the surface in the case of the CGD system. The carbon quantity after 1 mo is higher in the decanol-treated sample, and this could be related to decanol integration inside the chemical garden structures.

We then analyzed specific surface structures of each system after 1 d and 1 mo of growth. Images are reported in *SI Appendix*, Fig. S3. The surface of the CG system after a 1-d incubation time shows a smooth layer of Na2SiO3 with NaCl crystals. The surface of the CGD system (also 1 d incubation time) shows instead a smooth layer of Na2SiO3 with some Na2CO3, NaCl, and CaSiO3 crystals. Some spots show double the amount of carbon compared to the rest of the sample (*SI Appendix*, Fig. S3*F*). This could correspond to spots where decanol was highly integrated. On the surface of systems left reacting for 1 mo (CG and CGD), we notice larger-scale structures compared with 1 d of incubation. In the case of the CG system structures, crystals of CaSiO3, NaCl, and CaCO3 are visible. On the surface of the CGD systems, we observe CaCO3, NaCl, and spots rich in Ca. Here again, we find spots with a high level of carbon that can be correlated to decanol integration (*SI Appendix*, Fig. S3 *K* and *M*). All the EDXS data confirmed the XPS previous conclusions. The increase of dimension of surface aggregates is directly related to the ongoing reaction time of the system. We can conclude from this analysis that decanol integration is increasing over time and is specific to CGD systems.

#### B.1. Decanol integration in chemical garden structures.

As shown previously, when decanol is incubated with the sodium silicate system before calcium chloride addition, macroscopic-scale structures emerge that are different from typical chemical gardens. To understand whether decanol is integrated not only in the CGD samples surface, as implied by XPS and EDXS analysis, but also in the whole 3D CGD structures, we chose to perform a color-based assay. Decanol colored with Sudan Black B (2 mM) was layered on top of the silicate solution, and CaCl2 powder was added to it. Systems were incubated for 1 d. The newly formed structures integrated Sudan Black B and remained stained with its color even after three washes (*SI Appendix*, Fig. S4) in their whole structure. As negative control, CG systems in the presence of SBB were created, and the SBB did not stain the CG crystals when no decanol was present. The integration of the dye as a proxy for the largely nonpolar decanol, even on a short time scale, supports the integration of the decanol in the crystal garden structures and supports the results from the EDXS analysis.

### C. CGD Structure–Directed Membrane Assembly.

**Fig. 3.**
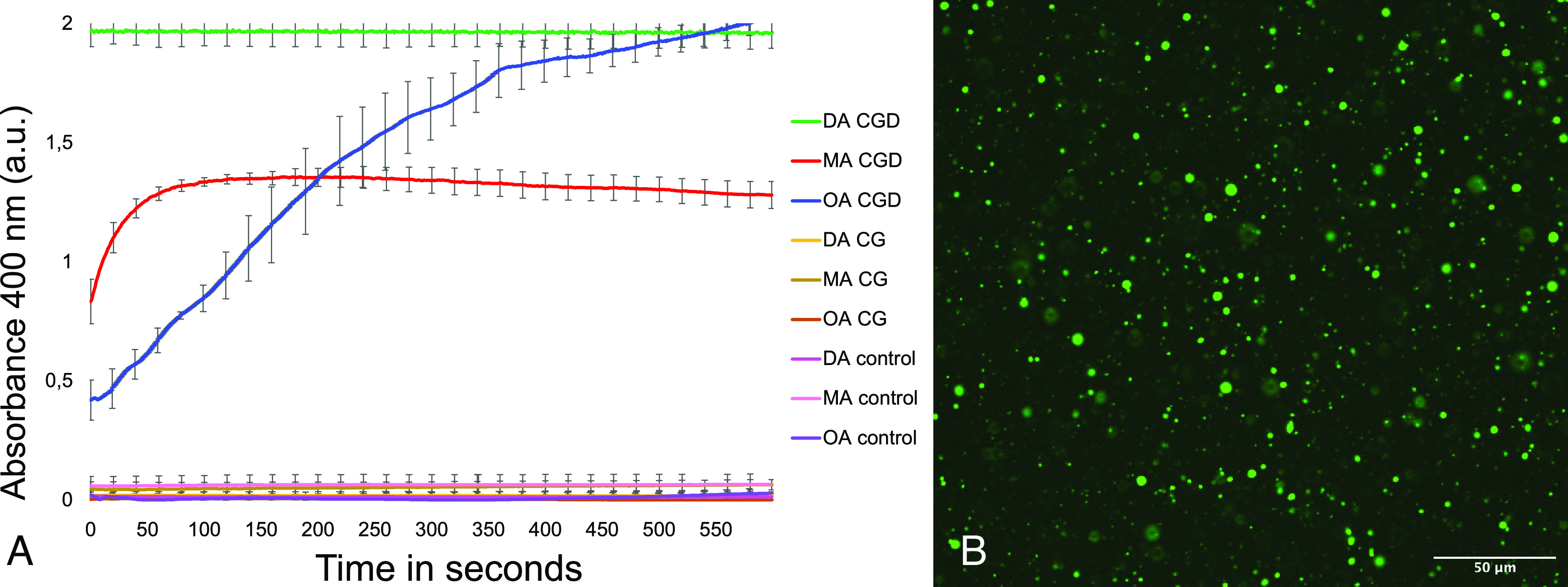
CGD crystal-directed vesicle assembly. Vesicle formation was monitored in real time using changes in absorbance values. First, 0.2 M bicine buffer was incubated alone (control) or with CG or CGD samples. Fatty acids/soaps decanoate (DA), myristoleate (MA), or oleate (OA) were then added to the buffered solutions (*A*). Readings were performed in triplicate, normalized over the weight of crystals introduced in each cuvette and also through pure bicine buffer signal subtraction. Absorbance was registered each second for 10 min, and SE is reported for each 20-s interval. In *B*, fluorescence imaging of decanoic acid CGD-induced vesicle formation. Size bar 50 microns.

Once decanol integration into the whole inorganic mineral structures was confirmed, we decided to test whether its presence could have supported fatty acid vesicle formation. We produced CG and CGD (1 mo) dried crystals, we cut small sections of them (∼20 mg), trying to preserve their structure, placed them on the bottom of a cuvette, and added bicine buffer (0.2 M pH 8.5). We afterward pipetted decanoate, oleate, or myristoleate micelles to each cuvette in the following concentrations: 50 mM, 1 mM, and 5 mM, respectively. These concentrations are slightly higher (20%) than the critical vesicle concentration (CVC) reported in the literature (47) for each of the fatty acids used here. To check for vesicle formation, the absorbance at 400 nm was measured, and the resulting kinetics are shown in Fig. 3*A*, *C*, and *D*. After the kinetics measure, CG and CGD crystals can still be visualized on the bottom of the cuvettes and their integrity seems to be conserved.

In the case of CGD systems, we observe absorbance variation with vesicle and micelle formation (see Fig. 3*A*, *SI Appendix*, Figs. S5 and S6). No absorbance variation or vesicles are instead noticed in the case of micelle addition to CG samples or to pure bicine buffer in this time frame. Decanoic acid shows an instantaneous increase of absorbance just after mixing with a stable high value; see Fig. 3*A*. This variation is related to supramolecular structure formation including membrane vesicles as confirmed with brightfield and fluorescence microscopy. Myristoleic and oleic acid show two different readouts, as can be seen in Fig. 3*A*, where the maximum level of absorbance is reached after a specific and different interval of time (1 min for myristoleic and 10 min for oleic acid). This is likely due to the different apparent pKa values for these fatty acids. We confirmed the presence of vesicles under these conditions using microscopy. The formation of vesicles under these conditions was further confirmed by a dye encapsulation test with 8-hydroxypyrene-1,3,6-trisulfonic acid (HPTS) addition to the bicine buffer. From the obtained images (Fig. 3*B* and *SI Appendix*, Figs. S5 and S6) we confirm vesicle formation for all three fatty acids. As an example, Fig. 3*B* shows clear encapsulation of HPTS inside membranes created using decanoic acid. We further analyzed vesicle formation using Merocyanine 540, a dye specifically sensitive to molecular packing. We observed that the incubation with CGD crystal lowers the critical vesicle concentration (CVC) of oleic, myristoleic, and decanoic acid. Data are shown in *SI Appendix*, Fig. S7. Furthermore, with respect to decanoic acid, CGD presence seems to shift the pH at which vesicles are formed, rendering feasible vesicle assembly at pH 8.5 (*SI Appendix*, Fig. S8). As a control, we studied vesicle formation and CVC with the buffer systems saturated with 1-decanol to determine whether the presence of a low soluble amount of decanol was responsible for the shift in CVC and vesicle formation. We determined that the presence of 1-decanol in solution was not sufficient to produce these results and that the presence of the CGD was necessary.

## 2. Conclusions

Many exploratory studies have shown that chemical gardens are a fertile and generative model system for prebiotic chemistry (48–51). They exhibit a broad range of nonlinear, pattern-forming chemical phenomena, the extent of which is still being discovered. While the relevance of hydrothermal vents and chemical gardens to the origins of life has long been recognized by the astrobiology community (3, 49, 52), the present work has taken steps by exploring how amphiphilic alcohols and acids—key components in protocell models—could have interacted with chemical gardens leading to the evolution of primitive cells.

We used silicate-based chemical gardens to implement an alkaline hydrothermal vent analogue. Integrating chemical gardens with a medium-chain alcohol, we generated a two-phase system where the alcohol was clearly separated from the silicate solution. The crystals localized at the interface showed random motion. Beneath the interface in the aqueous phase, new structures were created by the presence of decanol in the system. These new structures showed macroscopic and microscopic variability as well as compositional differences. As analyzed by XPS and EDXS, the materials comprising CG or CGD samples after 1 d or 1 mo of incubation appear similar in the overall atomic percentage. However, the observed structures are different. The integration of decanol on the inorganic surface changes the resulting morphology of the precipitates, and in the case of samples incubated with decanol, the decanol molecular interaction seems to increase salt aggregation upon the membrane surface, even if initially inhibiting the metal jets from the silicate surface. Furthermore, in the case of incubation with decanol, there are parts of the solid membranes which show an increased carbon presence. This likely correlates with decanol integration in the whole structure and that was confirmed through the Sudan Black B staining.

In conclusion, we demonstrated how medium-chain alcohols could have interacted with and evolved complex chemical garden structures. In a hydrothermal vent scenario, such structures could have aided the formation of supramolecular structures such as vesicles and micelles. These entities could have been composed of fatty acids exploiting fatty alcohol interaction. We in fact demonstrated that medium- (decanoic acid) and long-chain fatty acids (oleic and myristoleic), in the presence of chemical gardens formed in existence of decanol, are able to widely increase the creation of vesicles. These vesicles are generated even at concentrations close to the CVC, where their creation is normally minimal. We conclude that vesicles are formed and supported in our system only when the crystal garden is generated in the presence of decanol.

This work lays the foundation for the integration of organic compounds into silicate chemical gardens, including compounds that could have formed or helped vesicle formation such as fatty acids and fatty alcohols. Further studies could be performed on the interaction of other molecules such as DNA, amino acids, and enzymes inside hydrothermal vent–like structures and their compartmentalization into vesicles. This would lead to the creation of new experimental models that explore the interaction of inorganic and organic systems in the origin of life studies.

## Materials and Methods

### A. Materials.

The following reagents were supplied by Merck Life Sciences: 1-decanol (150584), sodium silicate solution (338443), Sudan Black B and calcium chloride (anhydrous, granular, ≤7.0 mm, ≤93.0% C1016), decanoic acid (W236403), and myristoleic acid (M3325). Oleic acid was supplied from TCI Europe N.V. (112-80-1). Milli-Q water was obtained using PURELAB flex 2 ELGA LabWater. Tubes were provided by ROTILAB and 6-well plates from Corning Incorporated Costar. Cuvettes (01938-00) were provided from KARTELL SPA.

### B. Vertical Observation System.

Sodium silicate and Milli-Q water were mixed to obtain a 2.9 M solution at pH 11.5. This solution was placed inside glass vials of 12-mm diameter (Rotilab glass centrifuge tubes). Inside each glass vial were added in total 5 mL of solution: 5 mL of sodium silicate solution (Fig. 1*A*) or 4 mL of sodium silicate solution and 1 mL of 1-decanol (Fig. 1 *B* and *C*). Where the alcohol phase was introduced, 1 mL of 1-decanol was carefully layered on top of the silicate phase or mixed with it. Then, 300 mg calcium chloride seeds were dropped on the top of the solution manually. Systems were observed from the side and their evolution recorded over 2 h. The system was tested five times and repeatability confirmed. All the videos were recorded using C920 Logitech HD Pro.

### C. Horizontal Observation System.

First, 5 mL of 2.9 M Na2SiO3 solution was added to each well of a 6-well plate (Corning Incorporated Costar 6-well cell culture plate, flat bottom with a lid, tissue culture–treated nonpyrogenic polystyrene). In the experiments where fatty alcohol was introduced, 3 mL 1-decanol was carefully added on top of the aqueous phase (the minimum amount required to totally cover the sodium silicate phase). In the case of the 1-decanol integration control, decanol was colored through the addition of Sudan Black B (2 mM). Then, using a Carver hot press, 300 mg calcium chloride seeds were smashed, placed upon parafilm, and manually dropped in each well. Evolution of the system was recorded from above during the first 2 h, and images of the system were taken after 2 h. The system was tested five times and repeatability confirmed. In the case of samples left reacting 1 mo, the plate was sealed to avoid evaporation. As with the vertical observation system, all the videos were recorded using the C920 Logitech HD Pro webcam and all the Images taken using an iPhone 8 webcam and analyzed using Fiji. Images were inverted and pixel density maps created using Fiji’s plot profile function. The mean pixels from an empty well were subtracted from the experimental values as background.

### D. X-ray Photoelectron Spectroscopy.

Sections of sodium silicate solution left reacting without (CG) and with decanol (CGD) were obtained from the surface of samples left reacting for 1 d in the horizontal system; the remaining liquid part was poured away and the solid one washed five times with Milli-Q water and pure ethanol and left to dry in a vacuum chamber for 1 wk. Parts of the obtained solids surfaces were cut and attached to a silicon wafer through carbon tape. CG and CGD samples were analyzed using and XPS apparatus located in an UHV (Ultra High Vacuum) analysis chamber equipped with a Mgk X-Ray source (photon at 1253.6 eV) and a hemispherical electron energy analyzer VSW (PSP power supply). The maximum energy resolution of the apparatus is 0.86 eV. Characterizations have been carried out at medium (PE = 50 eV) and high (PE = 20 eV) resolution. Single core levels were analyzed using Voigt functions for lineshape deconvolution after subtraction of a Shirley background.

### E. SEM and SEM EDXS.

Sections of sodium silicate solution reacted without (CG) and with decanol (CGD) were obtained from samples after 1 d or 1 mo in the horizontal system. The liquid part was poured away, and the solids were washed five times with Milli-Q water and left drying for 1 wk in a vacuum chamber. For each condition, three sections of the solid were cut and attached to a supporting stub and coated with Au or C depending on the type of analysis performed (SEM or EDXS respectively). A Jeol JSM-7001F SEM-FEG machine equipped with an Oxford INCA PentaFETx3 EDXS detector was used for morphological image acquisition and compositional analysis. SEM images were acquired using Secondary and Backscattering Electrons. SEM and EDXS analysis were performed at 15-keV beam energy and 10 mm of working distance.

### F. Surface Directed Membrane Assembly.

Oleic acid, decanoic acid, and myristoleic were added to a diluted NaOH solution to a final 250 mM concentration and mixed few minutes by vortex. Sodium bicine 0.2 M was produced dissolving bicine in Milli-Q water and adjusting the pH to 8.5 using NaOH 5 M. CG and CGD samples left reacting 1 mo were washed 5 times using Milli-Q and dried over 1 wk. Solids were afterward cut, leaving their surface as intact as possible and added to a plastic cuvette. Inside each cuvette, we tried to load a mineral crystal of ∼20 mg, and all the data are normalized over this standard weight. Fatty acids solutions were added to each cuvette to obtain a concentration of ∼20% higher than the CVC and mixed by pipetting. Final concentrations of decanoate, oleate, or myristoleate micelles were, respectively, 50 mM, 1 mM, and 5 mM. Kinetic measurements were run for 10 min, and absorbance value at 400 nm was recorded each second. Three replicates were analyzed, and data were normalized over the weight of crystals introduced on the bottom of the cuvette; background signal from pure bicine buffer with no fatty acid addition was subtracted. Vesicles formation was checked through the addition in each cuvette of HPTS (2 mM final concentration). After 10 min of incubation, the solutions were diluted 1:30 in dilution buffer (0.2 M bicine buffer pH 8.5, CVC of each corresponding acid, and 12% total volume of 1M glucose; this composition was specifically adapted to obtain a similar osmolarity in the dilution buffer to the buffer where the vesicles formed). Afterward, 70 μL of solution was charged in each well of 96-well Plate cell titer Ultra and visualized using the Nikon SIM with AX confocal microscope. The CVC was analyzed using merocyanine 540 (MC 540) addition in the presence of CG, CGD, and decanol. MC540 was dissolved in a 1:1 ethanol/water mix (1 mg/mL). Bicine buffer 0.2 M in Milli-Q was adjusted to pH 8.5 using NaOH. Phosphate buffer 0.1 M was prepared similarly. To 10 mL of buffer, 33 μL MC540 solution was added. Buffers were incubated with decanol, CG, and CGD, poured away after 10 min, and centrifuged at 13,000 rcf for 3 min. The obtained supernatants were used to run the CVC titrations. Absorbance values were obtained using the Spark Tecan plate reader and Corning transparent 96-well plates.

## Supplementary Material

Appendix 01 (PDF)

## Data Availability

All study data are included in the article and/or *SI Appendix*.
