# Supplementary material for: Hybrid organic–inorganic structures trigger the formation of primitive cell-like compartments
Source: Proc Natl Acad Sci U S A. 2023 Aug 10;120(33):e2300491120. doi: 10.1073/pnas.2300491120 (PMC10438843; doi:10.1073/pnas.2300491120)
Supplement: Supplementary file 1 — Appendix 01 (PDF) [file pnas.2300491120.sapp.pdf]

PNAS

Supporting Information for

Hybrid organic-inorganic structures trigger the formation of primitive cell-like compartments

S. Holler, S. Bartlett, R.J.G. Löffler, F. Casiraghi, C.I. Sainz Diaz, J.H.E. Cartwright and M.M. Hanczyc

Silvia Holler.
E-mail: silvia.holler@unitn.it

This PDF file includes:

Figs. S1 to S8

1. Supplementary Figure 1

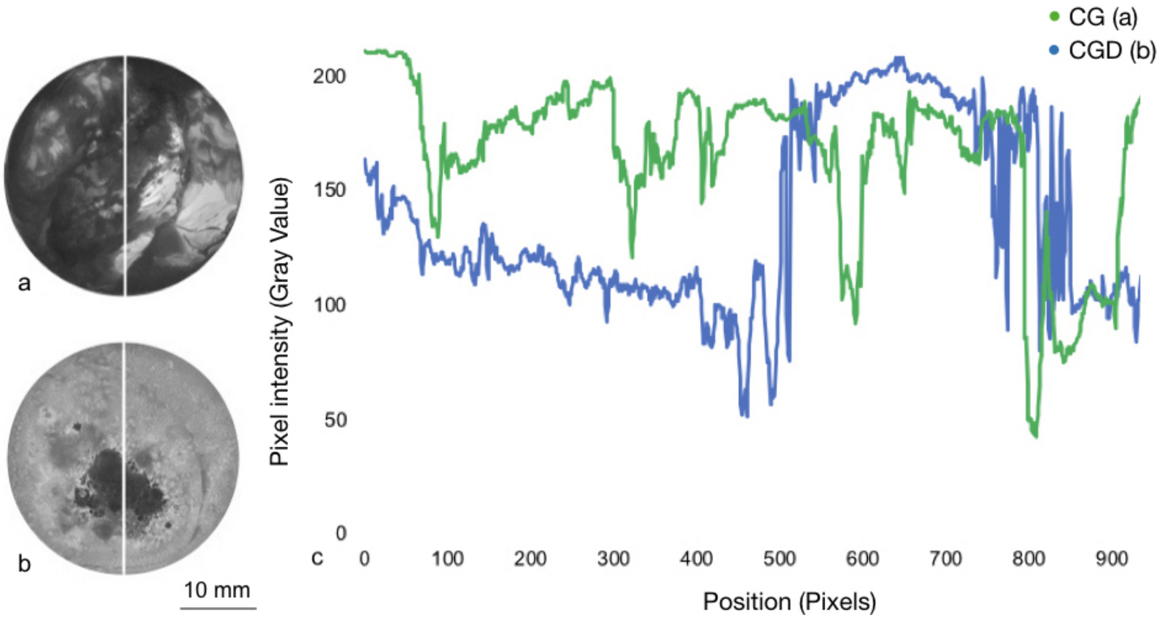

Fig. S1. Horizontal systems pixel intensity analysis. a) CG system (see also Figure 1d). b) CGD system (see also Figure 1e). c) Pixel intensities plot for the grayscale corresponding to the white lines on a and b images. Pixel plots look markedly different between CG and CGD samples. In CGD we observe that there is a region of high intensity (515-829 pixels) surrounded by a lower background. This region corresponds to the main body of the mineral salt from which a 'stardust' like structure is generated. This region is also the part of the experiment that shows random motion (see also Video 2). In contrast, the mean pixel intensity of the CG system is higher than the 'stardust' part of CGD (166 vs 135) and the profile shows a significant degree of gray intensity variation that corresponds to full and empty parts of the garden (these are absent in the CGD system).

2. Supplementary Figure 2

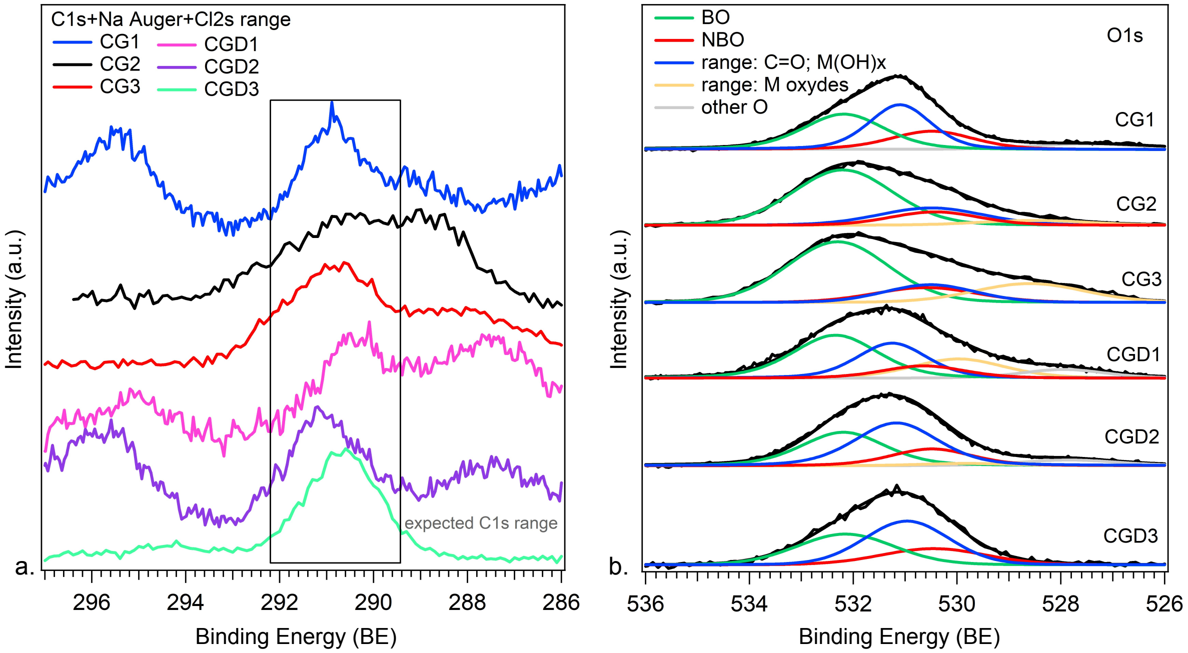

Fig. S2. XPS short range analysis for C1s and O1s. a. Comparison of CGs and CGDs samples after short range C1s Binding Energy (BE) alignment. b. Lineshape fitting for O1s short range BE alignment of CGs and CGDs samples

11 ## 3. Supplementary Figure 3

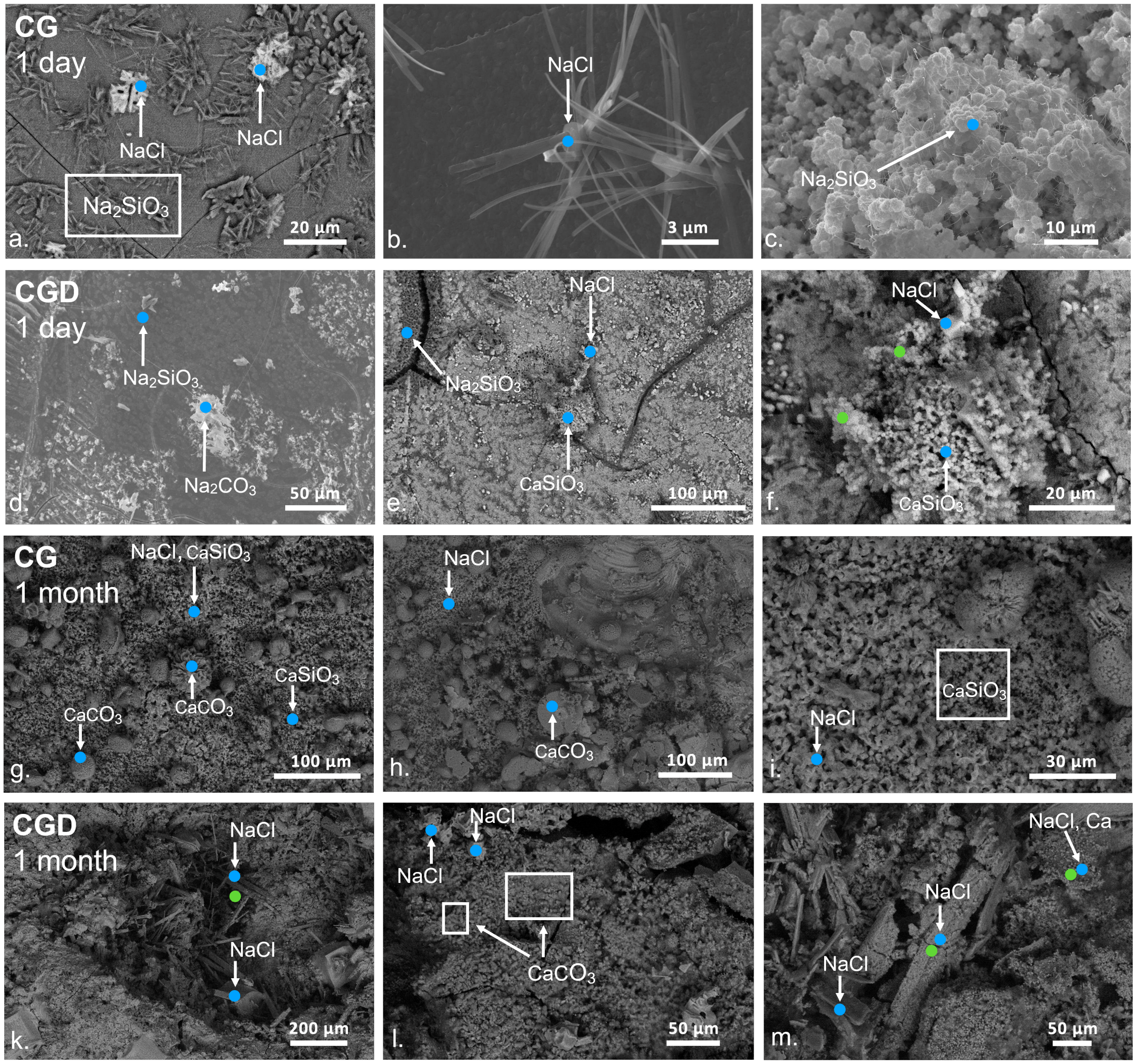

Fig. S3. EDXS small scale analysis. For each type of surface incubated for either 1 day (CG: a-c, CGD: d-f) or 1 month (CG: g-i, CGD: k-m), we marked squares with their specific area composition and blue dots with their point composition. The green dots correspond to spots that have double the amount of carbon compared to the rest of the sample. This could correspond to spots where decanol was highly integrated.

4. Supplementary Figure 4

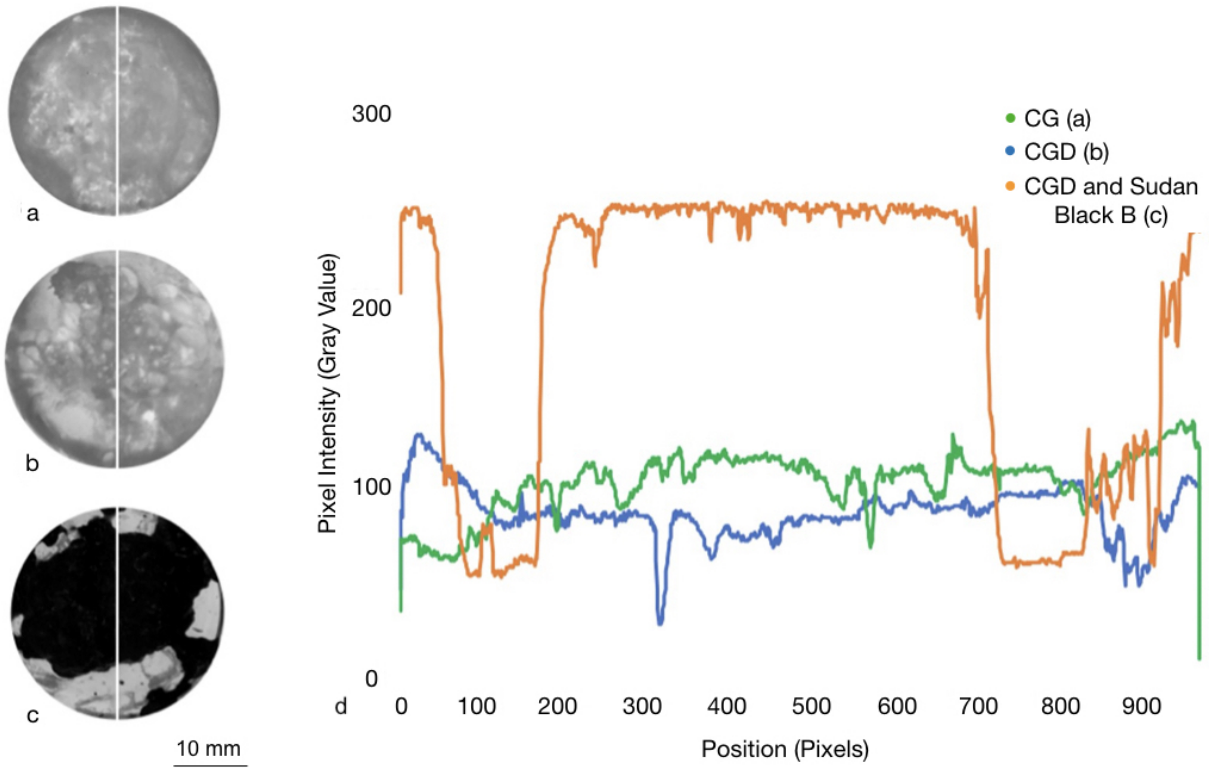

Fig. S4. Decanol integration in chemical gardens. Horizontal systems were created similarly to the ones described in Figure 1 and analyzed through pixel grayscale intensity analysis. a) CG system, b) CGD system, and c) CGD system where decanol colored with Sudan Black B (2 mM) was used. Images of the system were taken after 24 hours and 3 washes. d) Pixel intensities plot for the grayscale corresponding to the white lines on a, b and c images. Pixel intensity analysis indicates decanol integration inside the chemical garden structure c (200-700 pixels region) already after one day.

5. Supplementary Figure 5

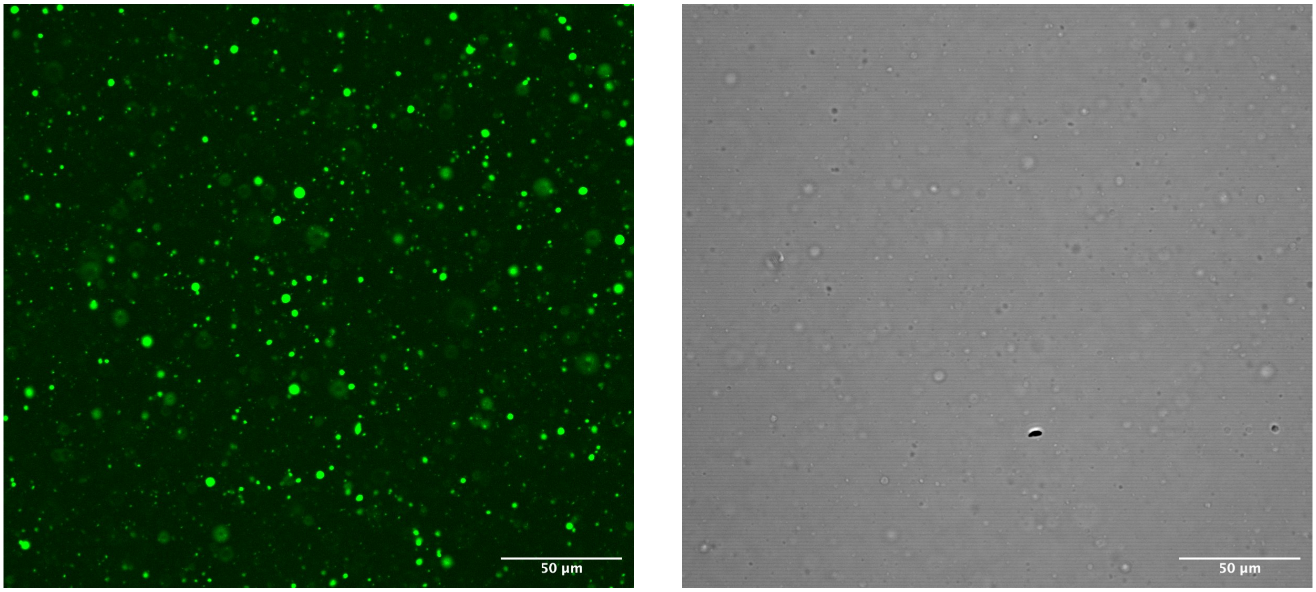

Fig. S5. Fluorescence (left) and brightfield (right) images of decanoic acid vesicles encapsulating HPTS. Vesicles were obtained after the addition of decanoate micelles solution (50 mM final concentration) to bicine buffer in a cuvette in contact with CGD sample. The image was taken 10 minutes after the mixing. The creation of vesicles can be visualized through HPTS integration.

6. Supplementary Figure 6

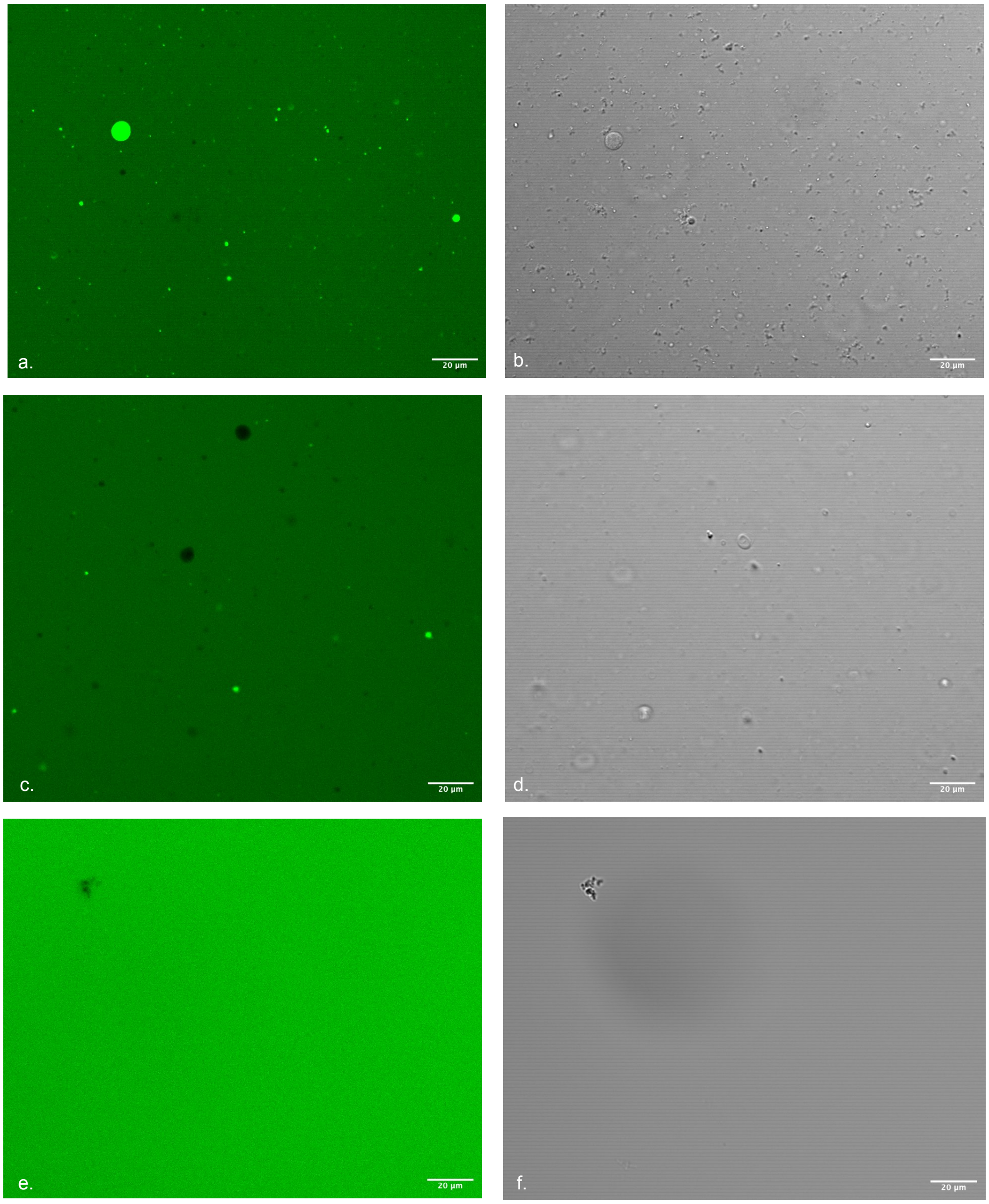

Fig. S6. Fluorescence (left) and brightfield (right) images of vesicles encapsulating HPTS. a and b) myristoleic acid vesicles and droplets, c and d) oleic acid vesicles and droplets. In all four images small, vesicles but also oil droplets, and small mineral crystals are visible. e and f) negative control upon oleic acid addition to CG samples. Only one crystal is visible and no HPTS integration can be visualized. Myristoleic acid and decanoic acid addition to CG system behaved similarly.

7. Supplementary Figure 7

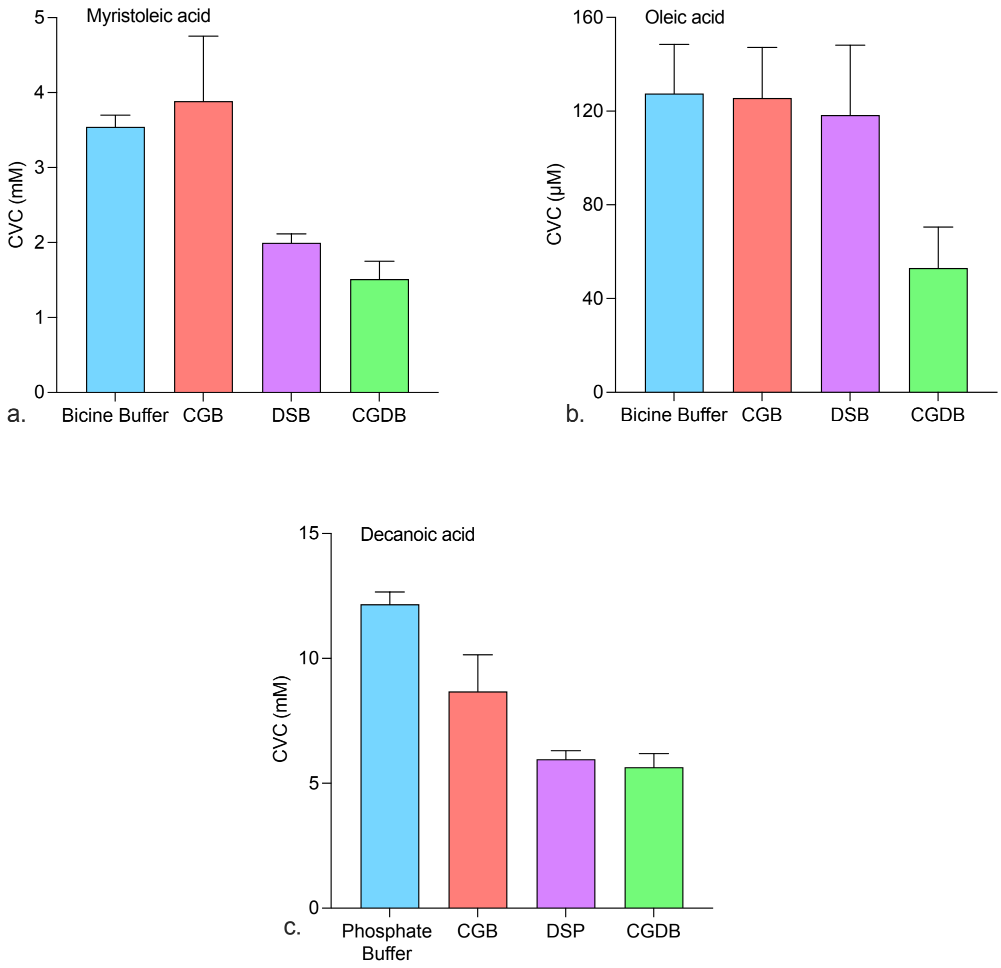

Fig. S7. CVC of Myristoleic, oleic and decanoic acid in the presence of CGs, decanol or CGDs. a. Myristoleic acid CVC analysis using bicine buffer, CGB (CG bicine), DSB (decanol saturated bicine) and CGDB (CGD bicine) b. Oleic acid CVC analysis using CGB, DSB and CGDB c. Decanoic acid CVC analysis using phosphate buffer, CGB (phosphate), decanol saturated phosphate (DSP) and CGDB (phosphate). Each condition was tested in three technical replicates and performed in experimental triplicates.

8. Supplementary Figure 8

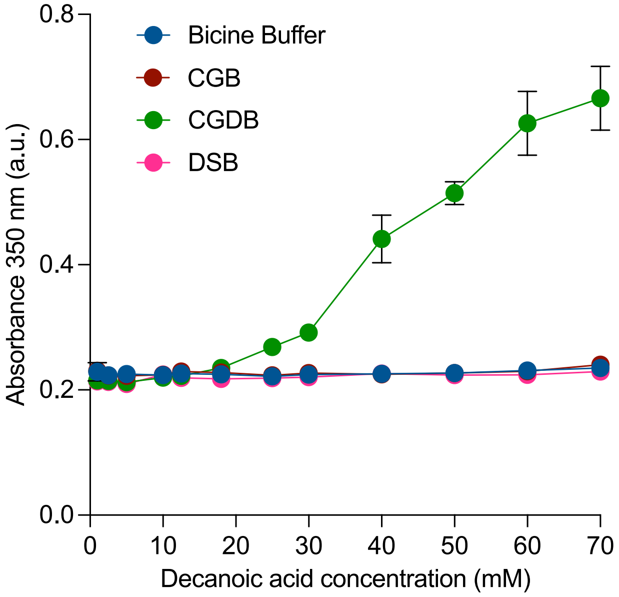

Fig. S8. Absorbance variation of decanoic acid in the presence of CGs, decanol or CGDs. Decanoic acid was added in different concentrations (from 0 to 70 mM) to bicine buffer, bicine buffer incubated with CG (CGB), bicine incubated with CGD (CGDB) and decanol saturated bicine buffer (DSB). Experiments were performed in triplicates.

9. Supplementary Video S1

Video S1. https://youtu.be/ck807PuiRj8_

10. Supplementary Video S2

Video S2. https://youtu.be/f_pxXHHHEoM
